# Effect of injected dexamethasone on relative cytokine mRNA expression in bronchoalveolar lavage fluid in horses with mild asthma

**DOI:** 10.1186/s12917-019-2144-x

**Published:** 2019-11-06

**Authors:** Stephanie L. Bond, Jana Hundt, Renaud Léguillette

**Affiliations:** Faculty of Veterinary Medicine, University of Calgary, Calgary, AB Canada

**Keywords:** Allergic asthma, Th-2 response, Th-17 response, REST analysis, Glucocorticoid therapy, Lung inflammation

## Abstract

**Background:**

Mild equine asthma is a common inflammatory airway disease of the horse. The primary treatment of mild equine asthma is corticosteroids. The purpose of this study was to investigate the effects of injected dexamethasone on relative IL-1β, IL-4, IL-5, IL-6, IL-8, IL-10, IL-12p35, IL-17, IL-23, IFN-γ, Eotaxin-2 and TNF-α mRNA expression in bronchoalveolar lavage (BAL) fluid in healthy Thoroughbred horses (*n* = 6), and those with mild equine asthma (*n* = 7).

**Results:**

Horses with mild equine asthma had a significantly greater bronchoalveolar lavage mast cell percentage than healthy horses both before and after treatment. Mild equine asthma was associated with a 4.95-fold up-regulation of IL-17 (*p* = 0.026) and a 2.54-fold down-regulation of IL-10 (*p* = 0.049) compared to healthy horses. TNF-α was down-regulated in response to dexamethasone treatment in both healthy horses (3.03-fold, *p* = 0.023) and those with mild equine asthma (1.75-fold, *p* = 0.023). IL-5 was also down-regulated in horses with mild asthma (2.17-fold, *p* = 0.048).

**Conclusions:**

Horses with mild equine asthma have a lower concentration of IL-10 in BAL fluid than healthy controls which concurs with human asthmatics. The marked up-regulation of IL-17 in horses with mild asthma suggests these horses had a true tendency of “allergic” airway inflammation in response to environmental allergens. Dexamethasone administration exerted anti-inflammatory effects associated with down-regulation of TNF-α in all horses, and decreased levels of IL-5 mRNA expression in horses with mild equine asthma. The inhibition of the Th-2 response, without any alterations to the airway cytology, indicates that maintained exposure to environmental allergens perpetuates airway inflammation.

## Background

Mild equine asthma, previously known as inflammatory airway disease, is a non-infectious inflammatory airway disease in the horse [1], which affects up to 66% of the equine population [2]. There is evidence that the Th-1, Th-2 and Th-17 immune responses are involved in the pathogenesis of various phenotypes of mild equine asthma, as indicated by multiple studies investigating the association between inflammatory cytokines and chemokines, and inflammatory bronchoalveolar lavage (BAL) cytology [3–6]. Recently a review proposed a “non-allergic equine asthma” phenotype [7], citing evidence which correlated a Th-1 response characterized by upregulation of IFN-γ mRNA in BALF-derived cells [3–5] with a generalized increase in BAL inflammatory cells. Furthermore, this review also linked this “non-allergic” phenotype with a Th-17 response, evidenced by increased IL-17 and IL-23 mRNA expression [3, 6], which is associated with a neutrophilic BAL. This terminology was expanded to another commonly occurring human asthma phenotype, with justification provided for the introduction of an “allergic equine asthma” phenotype [7] associated with a Th-2 predominant response, characterized by increased expression of IL-4 and IL-5 in BAL-derived cells [4, 6].

Inflammatory cells are recruited through increased production of inflammatory mediators, driven by increased activity of transcription factors. Logically, the efficacy of anti-inflammatory drugs, such as corticosteroids, has been evaluated in severe equine asthma through investigation of their impact on inflammatory gene expression in both BALF-derived cells [8–10] and bronchial epithelium [9]. Whilst there is a limited amount of clinical research on the efficacy of treatment on both airway hypersensitivity and hyperreactivity in horses with mild equine asthma [11], the impact of systemic corticosteroid administration on cytokine regulation in horses with mild equine asthma has not been investigated, and is the focus of the present study. Clarification of the cytokine responses in BALF-derived cells from horses undergoing treatment with dexamethasone would facilitate a greater understanding of the possible etiopathological pathways involved in mild equine asthma, and further elucidate how corticosteroids work to reduce inflammation in the lower respiratory tract of horses. Our hypothesis was that dexamethasone treatment alters cytokine gene expression in the lower respiratory tract of both healthy horses and those with mild asthma.

## Results

### Cytology results and enrolment

**Fig. 1:**
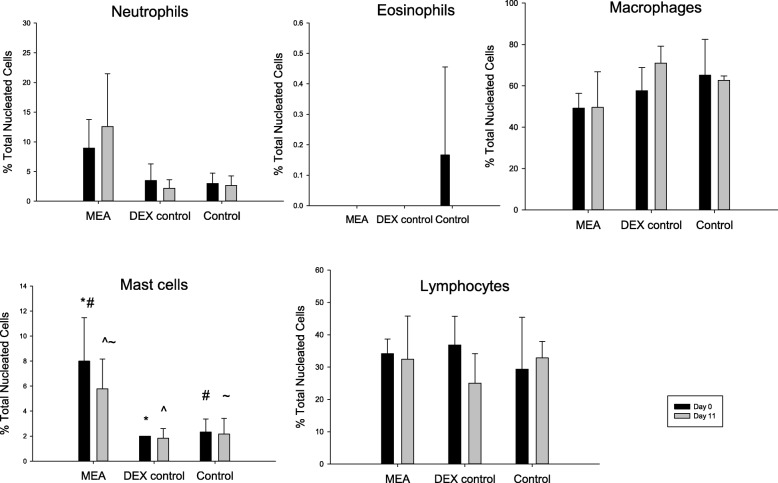
Bronchoalveolar lavage fluid differential cell counts (Mean ± SD) in 7 horses with mild equine asthma (MEA), and 3 healthy horses randomly allocated as a treatment control (DEX control), before (Day 0) and after (Day 11) treatment with 20 mg Dexamethasone IM SID. BALF differential cell counts for no treatment controls (n = 3, Control) are also provided. Superscript symbols indicate significant differences between categories with the same symbol (*P* < .05)

Of the seven horses enrolled in the MEA group; five horses had a mixed inflammatory profile (neutrophilic/mastocytic inflammation), and two horses exhibited mastocytic inflammation (Fig. 1). Six horses were classified as healthy based on their BAL cytology and clinical examination; three were randomly enrolled in the DEX group and received dexamethasone for 10 days and three were enrolled in the CONTROL group. Bronchoalveolar lavage fluid differential cell counts (±SD) for each group on day 0 and day 11 are shown in Fig. 1.

After accounting for the treatment group, there was no significant difference in differential cell count for any cell type between day 0 and day 11. The MEA group had a significantly greater mast cell percentage than both the DEX control group (*p* = 0.003) and the CONTROL group (*p* = 0.004); there was no difference between the DEX control group and the CONTROL group (*p* = 0.80) (Fig. 1). Although the MEA group had a greater neutrophil percentage than both the DEX group and the CONTROL group at both timepoints, after accounting for timepoint, there was no significant difference in the neutrophil cell count between treatment groups (*p* = 0.051). There was no significant difference in any other cell count (lymphocyte, eosinophil or macrophage) between treatment groups.

### Relative gene expression – healthy versus mild equine asthma

Mild equine asthma was associated with a 4.95-fold up-regulation of IL-17 (*p* = 0.026) and a 2.56-fold down-regulation of IL-10 (*p* = 0.049) compared to healthy horses (combined CONTROL group and DEX control group) on day 0 (Table 1).

**Table 1:** Relative gene expression in the BALF of 7 horses with mild equine asthma compared to healthy controls on day 0. The normalization factor (calculated from multiple reference genes) has a value of 1, and genes of interest are either up-regulated or down-regulated, in association with lower airway inflammation. * indicates a significant difference (*p* < 0.05)

Gene	Expression	Regulation	Standard Error	95% C.I.	*P* value
IL-1β	1.15	DOWN	0.07–7.25	0.01–18.51	0.79
IL-4	1.96	UP	0.31–13.03	0.09–48.31	0.12
IL-5	1.29	DOWN	0.08–14.83	0.01–40.83	0.69
IL-6	3.97	DOWN	0.01–4.80	0.00–66.38	0.09
IL-8	1.96	DOWN	0.11–2.67	0.02–8.00	0.08
IL-10	2.56	DOWN	0.06–2.73	0.01–8.37	0.049*
IL-12	1.14	DOWN	0.18–3.74	0.04–9.59	0.72
IL-17	4.95	UP	0.24–74.33	0.01–1407.10	0.03*
IL-23	1.41	DOWN	0.14–3.39	0.01–7.63	0.42
IFN-γ	1.03	UP	0.15–12.15	0.04–80.73	0.97
Eotaxin-2	3.49	UP	0.31–35.10	0.12–2104.64	0.053
TNF-α	1.01	DOWN	0.32–3.41	0.07–8.24	0.99

### Relative gene expression – effects of time/dexamethasone

There was no significant change in relative expression levels of any gene investigated (IL-1β, IL-4, IL-5, IL-6, IL-8, IL-10, IL-12, IL-17, IL-23, IFN-γ, Eotaxin-2 or TNF-α) in the BALF from the CONTROL group between day 0 and day 11.

In the DEX control group, TNF-α was down-regulated in response to dexamethasone treatment 3.03-fold (*p* = 0.023) (Table 2). There was no dexamethasone treatment effect on relative expression of IL-1β, IL-4, IL-5, IL-6, IL-8, IL-10, IL-12, IL-17, IL-23 or Eotaxin-2 (Table 2). IFN-γ was not present in detectable quantities at either timepoint in this treatment group and is therefore not included in Table2.

**Table 2:** Relative gene expression in 3 healthy horses before and after treatment with 20 mg dexamethasone IM SID for 10 days. The normalization factor (calculated from multiple reference genes) has a value of 1, and genes of interest are either up-regulated or down-regulated in response to treatment. * indicates a significant difference (*p* < 0.05)

Gene	Expression	Regulation	Standard Error	95% C.I.	*P* value
IL-1β	2.33	DOWN	0.01–4.28	0.01–21.33	0.35
IL-4	1.56	DOWN	0.16–4.04	0.05–7.53	0.4
IL-5	2.56	DOWN	0.01–17.84	0.01–29.49	0.5
IL-6	1.35	UP	0.03–71.96	0.02–84.78	0.59
IL-8	1.89	DOWN	0.06–3.14	0.02–5.05	0.29
IL-10	2.63	DOWN	0.06–1.80	0.03–2.72	0.11
IL-12	2.00	DOWN	0.09–1.98	0.06–5.09	0.19
IL-17	1.75	DOWN	0.02–11.83	0.01–38.45	0.53
IL-23	3.70	DOWN	0.02–2.85	0.01–4.56	0.11
Eotaxin-2	1.75	DOWN	0.09–6.38	0.02–19.53	0.42
TNF-α	3.03	DOWN	0.07–1.12	0.05–3.44	0.023*

In the MEA group, IL-5 was down-regulated 2.17-fold in response to treatment (*p* = 0.048) (Table 3). Furthermore, TNF-α was also down-regulated 1.75-fold in response to treatment (*p* = 0.023) (Table 3). There was no dexamethasone treatment effect on relative expression of IL-1β, IL-4, IL-6, IL-8, IL-10, IL-12, IL-17, IL-23, IFN-γ or Eotaxin-2 (Table 3).

**Table 3:** Relative gene expression in 7 horses with mild equine asthma before and after treatment with 20 mg dexamethasone IM SID for 10 days. The normalization factor (calculated from multiple reference genes) has a value of 1, and genes of interest are either up-regulated or down-regulated in response to treatment. * indicate a significant difference (*p* < 0.05)

Gene	Expression	Regulation	Standard Error	95% C.I.	*P* value
IL-1β	1.09	UP	0.26–4.83	0.07–8.74	0.77
IL-4	1.82	DOWN	0.14–2.80	0.04–7.14	0.08
IL-5	2.17	DOWN	0.11–2.15	0.05–8.88	0.048*
IL-6	2.23	UP	0.30–16.06	0.06–78.66	0.08
IL-8	1.64	UP	0.45–6.16	0.19–16.62	0.06
IL-10	1.31	UP	0.26–7.82	0.12–36.95	0.44
IL-12	1.69	DOWN	0.15–2.90	0.07–6.04	0.12
IL-17	1.08	UP	0.04–25.16	0.002–382.68	0.92
IL-23	1.22	DOWN	0.30–2.50	0.11–7.32	0.41
Eotaxin-2	1.16	UP	0.04–42.07	0.001–282.09	0.84
TNF-α	1.75	DOWN	0.19–1.69	0.07–3.55	0.023*
IFN-γ	2.70	DOWN	0.01–10.68	0.01–22.52	0.21

## Discussion

This study reports the effects of systemic administration of an anti-inflammatory corticosteroid medication, injected dexamethasone, on inflammatory gene expression in BAL-derived cells from healthy horses, and those with mild equine asthma. Horses with mild equine asthma had a significantly greater percentage of mast cell percentage than healthy horses before and also after dexamethasone treatment. Horses with mild equine asthma had up-regulation of IL-17 (4.95-fold) and down-regulation of IL-10 (2.56-fold) compared to healthy horses. In all horses, treatment with injected dexamethasone was associated with down-regulation of TNF-α. Dexamethasone administration was also associated with down-regulation of IL-5 in horses with mild equine asthma.

An unavoidable limitation of this study was the small number of animals enrolled. Ideally, we would have had 4 different groups within this study, treated and untreated horses with the disease of interest – mild equine asthma – and treated and untreated healthy horses. Whilst there are ethical considerations for including a group of untreated animals with mild equine asthma, the welfare implications for this group would not be substantial due to the absence of labored breathing at rest. This inclusion was precluded by the limited number of horses available on the same property for this study. It is noteworthy that three healthy horses were not treated to control for the effect of time (environment) and stress of sampling on BAL inflammatory cytokine expression. However, the authors do acknowledge that three horses in each of the control groups (CONTROL and DEX) is a small number, and results obtained and presented in this manuscript should thus be interpreted with caution. Whilst recognizing this, the changes described make biologic sense, and the fact they were detected with such a small sample size makes it highly likely that this is a true representation. The technique used for statistical analysis, REST, discussed further below, provides a robust, reproducible, highly accurate technique with which to analyze low abundance gene expression in small sample populations [12, 13]. The authors also acknowledge that with a larger study population additional differences in cytokine expression might have been observed; whilst this was unavoidable it might have introduced type II error.

The aim of our study was to investigate changes in gene expression in response to treatment, therefore relative quantification based on relative expression of a target gene versus a reference gene was suitable for our purpose. Housekeeping genes are present in all nucleated cells, as they are required for basic cell survival, and provide an endogenous control. We therefore chose to use 4 house-keeping genes, which have been previously validated for cytokine expression studies in BAL fluid from horses with mild equine asthma [14]. The reliability of the quantification process is highly dependent on a valid data analysis technique. Parametric analysis is inadequate for this purpose. Whilst several mathematical algorithms have been developed to calculate relative expression ratios, they only allow for the determination of a single difference in transcription between one control and one sample. In contrast, REST analysis provides i) a value for variability in ratios of gene expression, ii) a statistical analysis to assess the significance of this variability, as well as iii) a standard error and iv) 95% confidence interval of the ratios [12]. REST uses validated statistical randomization algorithms and bootstrapping of data, comparing each Ct value for each gene of interest with each Ct value for every housekeeping gene; expression ratio results of the genes of interest are tested for significance using a Pair Wise Fixed Reallocation Randomisation Test, and are plotted using standard error (SE) estimation via a complex Taylor algorithm incorporated into the analysis program [12]. This is necessary to obtain standard errors and confidence intervals [12]. Whilst this often leads to large confidence intervals of the ratios, additional studies have been performed to confirm the adequacy of this technique compared to other mathematical models; REST analysis is superior in the evaluation of relative RT-qPCR analysis [13]. Furthermore, other methods do not provide standard deviation values, which also explains why previous studies have not provided expression ratios between states of health and mild equine asthma [3, 4]. While this technique provides a highly accurate and reproducible tool with which to analyse low abundance gene expression in molecular biology, only one other study has used REST analysis to analyze cytokine expression in BAL fluid from horses [6].

Airway cytology is used in clinics as an indicator of therapeutic success, however, a literature review shows that without environmental modifications, corticosteroid therapy alone fails to normalize airway neutrophilia, even after treatment periods of up to 6 months [10, 11, 15–17]. Whilst there was no significant difference (*p* = 0.051) in airway neutrophilia detected between healthy horses and those with mild equine asthma, it is likely that the absence of a difference is due to a type II error due to the unavoidable small sample size. A reduction in airway neutrophilia has been achieved by transferring horses to a low dust feed, with the addition of oral dexamethasone administration being associated with greater improvement [9]. In contrast to severe equine asthma, it could be hypothesized that a lesser degree of inflammation might be controlled with corticosteroid treatment only, even in the presence of suboptimal environmental conditions. However, the absence of an improvement in the mastocytic and mixed inflammatory BAL cytologic profiles of the MEA group in the present study after 10 days of dexamethasone treatment, without switching to a low-dust feed or other environmental modifications, dismisses such a hypothesis. Even for horses with mild asthma, poor environmental conditions have a greater impact than dexamethasone therapy, and if persistent, sustain lung inflammation. Furthermore, the benefit of using an intramuscular route of administration for dexamethasone treatment guarantees bioavailability [18]; the lack of cytological improvement was therefore not due to poor absorption.

The fact that environmental conditions were not changed avoided the potential for this to contribute as a confounding factor. The effect of environmental dust has been well described in horses with severe asthma, and the present study provides valuable information on the effects of treating horses with milder inflammation without changing environmental conditions. In reality, it is challenging for horse owners to decrease environmental dust and this study design therefore mimics current practice where horses with mild asthma are treated with corticosteroids without significant environmental changes.

A limitation that was considered during analysis was that the authors were unable to determine whether coughing increased or decreased in response to dexamethasone treatment, as no numerical cough data was recorded. However, standardized quantification of coughing in horses to assess the efficacy of treatments is challenging; whilst an objective method for recording and counting coughs has been reported [19], it was not utilized. We acknowledge that this is a limitation of the present study, however, it was not possible to implement due to horses remaining in their environment on the farm, which was preferable to transporting them to an indoor research stable facility, which might have induced additional airway inflammation, even in healthy horses [20–24]. However, a previous study showed that contrary to horse owners, who spend more time with their horses on a daily basis, clinicians would not be able to notice an improvement in the clinical condition of horses with severe asthma after corticosteroid therapy [25]. Whilst we asked the owner to monitor for coughing over the duration of the trial, this was not objectively measured; there is no reported frequently would an owner need to monitor their horses, nor for what duration, to be able to standardize a cough measurement without a microphone recording and analysis software [19]. Furthermore, whilst the consensus statement inclusion criteria requires that coughing be chronic (> 3 weeks) [1], due to limited access to the horses enrolled in the study, the authors believe the observed period of coughing of > 2 weeks was sufficient to determine chronicity of disease in this instance. As these horses had evidence of lower airway inflammation, and the focus of this study was on the immunomodulatory effects of dexamethasone treatment and not on treatment effects on performance or clinical signs, it was felt that the lack of objective data regarding coughing was insufficient justification to preclude their enrollment in the study.

The substantial age range of the enrolled horses in the study (5–27 years) was also considered as a potential confounding factor in this study. Immunosenescence - age-related increases in pro-inflammatory cytokines - has been reported in horses, with healthy aged horses having increased mRNA expression of IL-6, IL-8, IFN-γ and TNF-α in plasma [26]. However, age related changes appear more tightly regulated in the lungs than in the systemic circulation [27]; there are no reports of age-related trends in BALF cytologic profiles in horses with mild equine asthma.

In human asthma, there is accumulating evidence which suggests that IL-17 production plays a key role in severe forms of asthma [28]. Since our results are based on mRNA, which can undergo post-transcriptional regulation and might therefore not reflect true protein concentrations, the presence of a 4.95-fold up-regulation of IL-17 is significant and might reflect horses with a true tendency of “allergic” airway inflammation in response to environmental allergens. IL-17 is an inflammatory cytokine involved in the recruitment and proliferation of neutrophils. A Th-17 response has been implicated in neutrophilic mild equine asthma, with an association between the BALF neutrophil ratio and increased IL-17 and IL-23 mRNA expression [3, 6].

Interestingly, in comparison with healthy horses, those with mild equine asthma also had a 2.56-fold down-regulation of IL-10. IL-10 is the intrinsic physiologic mechanism that inhibits pro-inflammatory cytokine synthesis [29]. In healthy human lungs, alveolar macrophages and circulating monocytes are the main sources of IL-10 [30]. In human BAL fluid, alveolar macrophages constitute > 80% of cells present; Th1/Th2 lymphocytes, cytotoxic B cells, B lymphocytes and mast cells comprise less than 10% of the cellular population [29]. Only one previous equine study has investigated IL-10 in horses with mild equine asthma, and it failed to detect a significant change with disease [6]. However, in agreement with the findings of the present study, the concentration of IL-10 in BAL fluid of human asthmatic patients is lower than in healthy controls, and an inverse association between asthma severity and IL-10 concentration has been established [29]. The absence, or reduced concentration of IL-10, associated with asthma enables the continued secretion of pro-inflammatory cytokines that contribute to lower airway inflammation, including IL-6, IL-5, IL-4, TNF-α, and IL-1.

In horses with mild asthma, we observed a down-regulation of IL-5 in response to dexamethasone administration. It has been shown that IL-5 is upregulated in horses with mild equine asthma, in both mastocytic and neutrophilic phenotypes [6]. In humans, IL-5 is highly specific for eosinophilic inflammation, and antibodies which block IL-5 actions are effective in reducing eosinophilic inflammation and airway hyperresponsiveness [31]. Whilst eosinophils are less commonly detected in equine BALF, excepting a sub-group of MEA reported predominantly in young horses associated with dust exposure [32, 33], it appears that environmental allergens are associated with both the clinical signs and lower airway inflammatory pathology observed in horses with MEA [7]. Furthermore, while not a focus of the present study, corticosteroid administration has been shown to reduce both airway hypersensitivity and hyperreactivity in horses with MEA [11]. In mice, a single antigen challenge has been shown to increase IL-5 protein and mRNA in BALF and lung tissue, with dexamethasone treatment reducing both airway hyperresponsiveness, and IL-5 mRNA in BALF [34]. Consistent with this, the down-regulation of IL-5 observed in response to dexamethasone treatment in horses with MEA indicates a shift away from a dysregulated Th-2 response after allergen exposure.

Treatment with injected dexamethasone was also associated with down-regulation of TNF-α in all horses. In humans, it is acknowledged that TNF-α plays an important role in allergic inflammation of the bronchus, with increased levels of expression being reported in the serum of patients with allergic asthma in acute attack, compared to healthy individuals or asthmatics in clinical remission [35]. Similarly, TNF- α expression is increased in horses with mild equine asthma [3–5]. Furthermore, a week of oral glucocorticoid administration decreases serum TNF-α levels following an allergic asthma attack [36]. As glucocorticoid administration exerts an anti-inflammatory effect and is capable of decreasing TNF-α levels, it is therefore logical that there was a larger anti-inflammatory effect exerted on horses experiencing airway inflammation.

## Conclusions

Horses with mild equine asthma have a lower concentration of IL-10 in BAL fluid than healthy controls, which concurs with human asthmatics; the possible inverse association between equine asthma severity and IL-10 concentration warrants further investigation. The marked up-regulation of IL-17 in horses with mild asthma suggests these horses had a true tendency of “allergic” airway inflammation in response to environmental allergens. Dexamethasone administration exerted anti-inflammatory effects associated with down-regulation of TNF-α in all horses, and decreased levels of IL-5 mRNA expression in horses with mild equine asthma. The inhibition of the Th-2 response, without any alterations to the airway cytology, indicates that while dexamethasone administration can help to reduce airway hypersensitivity and hyperreactivity, maintained exposure to environmental allergens perpetuates airway inflammation.

## Methods

### Animals and study design

This was a prospective, randomized, controlled clinical trial. The procedures performed on the horses enrolled in the present study have been previously published in an investigation of the upper and lower respiratory microbiota of the horse, associated with both health and mild equine asthma [37]. Briefly, BAL fluid was collected from a herd of 13 deconditioned Thoroughbred horses (geldings; 5–27 years old; weights not obtained) used for Chuckwagon racing. Horses resided on a single property (Okotoks, AB, Canada) were kept outside in dirt paddocks, and were released to the owner at the conclusion of the trial. Some horses had an owner-reported history of chronic coughing (> 2 weeks, *n* = 7), which while not standardized or objectively counted [19], was determined to be an abnormal finding in the owner’s assessment. As owners spend a considerable amount of time with their animals, they are skilled observers at noticing airway disease in horses [25]. It has been suggested that more emphasis be placed on owner-assessment than on clinical, cytological and endoscopic examinations, performed by veterinarians who examine the horses infrequently [25]. Furthermore, owners are better able to determine whether treatment is effective or not, than veterinarians [25]. Coughing was confirmed by the investigators in these horses (*n* = 7) via observation or elicited through tracheal palpation. In contrast, no cough could be elicited in healthy horses. With the exception of coughing, horses were judged to be clinically healthy based on thorough physical examination (performed by RL). They were fed a diet of second-cut alfalfa hay for the duration of the trial, beginning a minimum of 2 days before initial sampling. BAL were performed on all horses (*n* = 13) on day 0. On day 1, horses were allocated into one of three treatment groups based on their BAL cytology, history and clinical examination (mild equine asthma versus healthy) and random selection (among healthy horses); MEA (horses with mild equine asthma; *n* = 7), DEX (healthy horses treated with dexamethasone; *n* = 3) and CONTROL (healthy horses not treated with dexamethasone; n = 3). Horses were considered to have mild equine asthma based on the following inclusion criteria (defined in a consensus publication [1]): 1. a BAL with increased percentage of mast cells (> 2%) or/and eosinophils (> 0.5%) or/and neutrophils (> 10%), 2. owner-reported chronic coughing, confirmed via observation or tracheal palpation during clinical examination, and 3. absence of labored breathing at rest. Horses in MEA and DEX groups were then administered dexamethasone (20 mg, IM) every morning for 10 days. No other medications were given to horses for the duration of the trial. On day 11, the BAL procedure was repeated.

### Sampling procedure

Horses were pre-medicated with acepromazine maleate (0.07–0.08 mg/kg, IM/IV) approx. Thirty minutes prior to procedures. Horses were sedated to effect with xylazine hydrochloride (0.4–0.5 mg/kg, IV) and butorphanol tartrate (0.05–0.1 mg/kg, IV). A BAL was then performed as previously described [37]. Briefly, a balloon-tipped BAL tube (Mila International, SKU: BAL300) was inserted until wedged against the wall of a bronchus, and 2 boluses (250 ml/bolus) of sterile isotonic saline (0.9% NaCl) solution were sequentially instilled. Lavage fluid was recovered and two 10 mL aliquots were immediately stored at 4 °C. A differential cell count was performed within 6 h of sample collection and was performed on a minimum of 400 cells [38]; epithelial cells were not included in the differential count. Preparation of slides was performed with 400 μL of BAL fluid, which was centrifuged using a Cytospin (90 x g for 5 min) and stained with modified Wright-Giemsa stain. Two 50 mL aliquots of BAL fluid were centrifuged at 700×g for 10 min; the supernatant was then discarded, and the cell pellets resuspended in 1.5 mL of RNAlater (Qiagen, Mississauga, Ontario, Canada). Samples were stored at − 80 °C until RNA extraction.

### RNA extraction, cDNA synthesis and qPCR analysis

Total RNA was extracted using the RNeasy Mini Kit (Qiagen, Mississauga, Ontario, Canada), using 40 μL RNase-free water to elute samples; the initial eluate was re-applied directly to the spin column membrane and centrifuged at 8000 g for 1 min. The quantity and quality of the extracted RNA were measured using the Nanodrop (ND 1000) spectrophotometer. Contaminating genomic DNA was removed prior to cDNA synthesis using dsDNase (Thermo Scientific, #EN0771, Wilmington, DE, USA). Approximately 500 ng total RNA was retro-transcribed with the Omniscript® Reverse Transcription Kit (Qiagen, Mississauga, Ontario, Canada), as per manufacturer instructions, with RNaseOUT (Thermo Scientific, Wilmington, DE, USA) and Oligo (dT) primers (Invitrogen, Burlington, Ontario, Canada) included in the reaction mixture. Primer sequences used for IL-1β, IL-4, IL-5, IL-6, IL-8, IL-10, IL-12, IL-17, IL-23, IFN-γ, Eotaxin-2 and TNF-α have been previously described [3, 6, 39] (Table 4). Reference genes included GAPDH, SDHA, HPRT and RPL-32, which have been shown to provide accurate normalization for gene expression studies in BALF from horses with mild equine asthma, treated with dexamethasone [14]. Amplification of target RNA was in 25 μL total reaction volume containing 13 μL PerfeCta® SYBR® Green SuperMix, Low ROX™ (Quanta Biosciences), 50 nM (Eotaxin-2 and IFN-γ) and 100 nM (all other genes) forward and reverse gene-specific primers, 4 μL nuclease-free H_2_O, and was completed by adding 4 μL of cDNA template. Amplification was performed in 96-well skirted qPCR plates (VWR 82006–704) in a thermal cycler (BioRad CFX96 Touch™ Real-Time PCR Detection System). The reaction was initially denatured at 95 °C for 3 min, which was followed by 45 cycles of 15 s denaturation at 95 °C, and 30s annealing at 62 or 64 °C (gene specific; Table 4). Fluorescence data acquisitions occurred at the end of each annealing cycle. A final melt curve analysis was run from 60 to 90 °C at .5 °C increments for 5 s, with a fluorescence data acquisition after each step. Reactions were executed in triplicate, with template from samples collected on day 0 and day 11 from the same horse included on the same plate. No RT and negative controls were included on each plate. Cycle threshold (Ct) values were generated from Bio-Rad CFX Manager 3.1 software, with a user defined baseline threshold of 1146.22 (genes with 62 °C annealing temperature) and 984.66 (genes with 64 °C annealing temperature).

**Table 4:** Oligonucleotide primer sequences for amplification of target equine genes

Gene	Oligo	Sequence (5′-3′)	PCR Product size (bp)	Annealing temperature (°C)	Sequence accession number(s)	Reference(s)
GAPDH	Forward	GGTGAAGGTCGGAGTAAACG	106	64	AF157626; AF083897	Beekman, 2011
	Reverse	AATGAAGGGGTCATTGATGG				
HRPT	Forward	AATTATGGACAGGACTGAACGG	121	62	AY372182	Beekman, 2011
	Reverse	ATAATCCAGCAGGTCAGCAAAG				
SDHA	Forward	GAGGAATGGTCTGGAATACTG	91	62	DQ402987	Beekman, 2011
	Reverse	GCCTCTGCTCCATAAATCG				
RPL-32	Forward	GGGAGCAATAAGAAAACGAAGC	138	62	CX594263	Beekman, 2011
	Reverse	CTTGGAGGAGACATTGTGAGC				
IL-1β	Forward	ACCATAAATCCCTGGTGCTG	179	64	D42147; U92481; D42165	Beekman, 2012
	Reverse	CGTCCCACAAGACAGGTACA				
IL-4	Forward	TCGTGCATGGAGCTGACTGTA	151	64	L06010; AF035404	Beekman, 2012
	Reverse	GCCCTGCAGATTTCCTTTCC				
IL-5	Forward	AAACTGTCCAAGGGGATGCT	169	64	U91947	Beekman, 2012
	Reverse	TCCGTTGTCCACTCAGTGTT				
IL-6	Forward	AGCAAGGAGGTACTGGCAGA	173	62	U64794; AF005227; AF041975	Beekman, 2012
	Reverse	CCTTTTCACCCTTGAACTCG				
IL-8	Forward	CGCACTCCAAACCTTTCAAT	165	62	AY184956; AF062377	Beekman, 2012
	Reverse	TCAAAAACGCCTGCACAATA				
IL-10	Forward	ATCGATTTCTGCCCTGTGAA	174	62	U38200	Beekman, 2012
	Reverse	CGTTCCCTAGGATGCTTCAG				
IL-12 p35	Forward	CATGAATGCCAAGCTGTTGA	185	64	Y11130	Beekman, 2012
	Reverse	AGGCATGAAGAAGGATGCAG				
IL-17	Forward	TATCGTGAAGGCGGGAATAG	210	62	EU744563	Beekman, 2012
	Reverse	TCCCAGATCACAGAGGGGTA				
IL-23	Forward	CTGGCCTGGAGTGCACATC	296	62	AY704416	Hughes, 2011
	Reverse	TTGTAGTCTCAGCATCTCCCTCTTC				
IFN-γ	Forward	CTATTACTGCCAGGCCGCGTT	404	64	U04050; D28520	Giguère, 1999
	Reverse	TCCTCTTCCGCTTCCTCAGGTT				
Eotaxin-2	Forward	CCTGAGAGCCGAGTGGTAAG	152	64	ENSECAT00000023737	Beekman, 2012
	Reverse	TTCTTGGCAGCCAGATTCTT				
TNF-α	Forward	CTTGTGCCTCAGCCTCTTCTCCTTC	1385	64	M64087	Giguère, 1999
	Reverse	CAGCTGGTTGTCTGTCAGCTTC				

### Statistical analysis

The relative expression software tool (REST), which allows for correction for PCR efficiency normalization with multiple reference genes, was used for analysis, and has been previously validated [12, 40] and shown to be a powerful tool in the investigation of relative gene expression in BALF from horses with mild equine asthma [6]. Briefly, the REST software uses a P(H1) test for statistical analysis which represents the probability of the alternate hypothesis; that the difference between the “sample” and the “control” group is due only to chance. The hypothesis test performs 2000 random reallocations (“Iterations”) of “samples” and “controls” between the 2 groups and counts the number of times the relative expression on the randomly assigned group is greater than that of the sample data. Subsequently the expression ratio results of the investigated genes are tested for significance by a randomisation test, which accounts for multiple comparisons. In this study, “samples” referred to the post-treatment samples collected on day 11, and “control” referred to the pre-treatment samples collected on day 0; analysis was performed with horses separated by treatment group (MEA, DEX and CONTROL). Alternatively, when the effect of airway inflammation was examined, “sample” referred to the MEA group and “control” referred to horses with a normal BAL at day 0 (DEX and CONTROL groups). Normality of the distribution of the BALF differential cell counts were tested by a Shapiro-Wilk normality test. A two-way repeated measures ANOVA (controlling for treatment group and timepoint [Day 0 versus Day 11]) was used to assess differences in cell counts between groups. A *p*-value ≤ .05 was considered significant.

## Data Availability

The datasets used during the current study are available from the corresponding author on reasonable request.

## Abbreviations

BAL: Bronchoalveolar lavage
CONTROL: Negative control group
DEX: Dexamethasone control group
MEA: Mild equine asthma treatment group
REST: Relative expression software tool
